# Investigation into the characteristics, triggers and mechanism of apnoea and bradycardia in the anaesthetized platypus (*Ornithorhynchus anatinus*)

**DOI:** 10.1093/conphys/cou053

**Published:** 2014-12-05

**Authors:** J. W. Macgregor, C. Holyoake, P. A. Fleming, I. D. Robertson, J. H. Connolly, K. S. Warren

**Affiliations:** 1 College of Veterinary Medicine, School of Veterinary and Life Sciences, Murdoch University, 90 South Street, Murdoch, WA 6150, Australia; 2 Environmental and Conservation Sciences, School of Veterinary and Life Sciences, Murdoch University, 90 South Street, Murdoch, WA 6150, Australia; 3 School of Animal and Veterinary Sciences, Charles Sturt University, Locked Bag 588, Wagga Wagga, NSW 2678, Australia; 4 Graham Centre for Agricultural Innovation, Locked Bag 588, Wagga Wagga, NSW 2678, Australia

**Keywords:** Apnoea, bradycardia, dive response, isoflurane, nasopharyngeal response, *Ornithorhynchus anatinus*, platypus

## Abstract

Health and conservation research on platypuses (*Ornithorhynchus anatinus*) may require anaesthesia to reduce stress and the risk of injury to both the animal and the researcher, as well as to facilitate examination and sample collection. Platypus anaesthesia can be difficult to manage, with reports of periods of apnoea and bradycardia described. This study investigated the conditions around sudden-onset apnoea and bradycardia in 163 field-anaesthetized platypuses as part of a health study. Anaesthesia was induced and maintained using isoflurane delivered in oxygen by face mask. Sudden-onset apnoea and bradycardia was observed in 19% of platypuses, occurring either at induction of anaesthesia, during recovery, or both. At induction, occurrence was more often recorded for adults (*P* = 0.19) and was correlated with low body temperature (*P* < 0.001), season (*P* = 0.06; greater incidence in summer) and longer pre-anaesthetic holding time (*P* = 0.16). At recovery, sudden-onset apnoea and bradycardia occurred only in platypuses that had been placed in dorsal recumbency as part of their examination, and correlated with poor body condition (*P* = 0.002), time in dorsal recumbency (*P* = 0.005), adults (*P* = 0.06), number of fieldworkers (*P* = 0.06) and females (*P* = 0.11). The sudden-onset apnoea and bradycardia we observed is likely to result from the irritant nature of isoflurane (stimulating the trigeminal nerve via nasal chemoreceptors). We propose that this mechanism is analogous to that of submersion of the face/nasal cavity in cold water during a natural dive response, but that the term ‘nasopharyngeal response’ would more appropriately describe the changes observed under isoflurane anaesthesia. Although we did not record any long-term adverse effects on platypuses that had undergone this response, the nasopharyngeal response could complicate the diagnosis of anaesthetic dose-dependent apnoea and bradycardia. Therefore, we suggest that these responses during anaesthesia of platypuses might be avoided by minimizing the stress around capture and handling, as well as reducing the time in dorsal recumbency.

## Abstract (precis)

By relating respiratory and heart rate changes observed in some platypuses under isoflurane anaesthesia to those produced by various stimuli to the trigeminal nerve we propose that in platypuses the changes that can result from isoflurane gas in the nasal cavity are most appropriately described by the term ‘nasopharyngeal response’.

## Introduction

Anaesthesia is an important field technique for platypus health and conservation research, facilitating sample collection and measurements while minimizing stress for the animal and reducing the risk of envenomation for the researcher (J. McKee, unpublished data). Apnoea and rapid-onset bradycardia, with heart rates as low as 12 beats/min during anaesthesia have been recorded in the platypus (Booth and Connolly, 2008; J. McKee, unpublished data). These changes have been reported to be self-resolving but to complicate the diagnosis of dose-dependent apnoea and bradycardia, which continue to deteriorate unless the dose is reduced. Apnoea and profound bradycardia under anaesthesia have also been reported in marine mammals and have been identified as the cause of anaesthetic deaths (McDonell, 1972; Gales and Burton, 1988; Phelan and Green, 1992). In both platypuses and marine mammals, these respiratory and cardiac changes under anaesthesia have been described as an inappropriate initiation of the dive response (Lynch *et al.*, 1999; J. McKee, unpublished data).

The dive response (or ‘dive reflex’ in older literature and some human literature; Foster and Sheel, 2005; Panneton, 2013) is a group of physiological changes that occur during submersion in water and has been described in a wide variety of aquatic and terrestrial vertebrate species (Irving *et al.*, 1941; Foster and Sheel, 2005; Panneton, 2013). The changes are broadly similar between species and consist of apnoea, bradycardia and compensatory redistribution of blood to the heart and central nervous system by peripheral vasoconstriction (Zapol *et al.*, 1979; Kooyman *et al.*, 1981; Foster and Sheel, 2005; Panneton, 2013).

Studies in a variety of mammalian species have investigated diving and the associated cardiovascular and respiratory effects, including apnoea, hypoxia, hypercapnia, contact with water, temperature change and increased external pressure on the body. While there are differences between species, some general observations have been made. Apnoea occurs due to a combination of conscious and reflex control (Butler and Jones, 1997; Foster and Sheel, 2005), where the reflex response is triggered by trigeminal nerve stimulation as a result of submersion of the face and/or nasal passages (‘facial submersion’) in water (particularly cold water; de Burgh Daly *et al.*, 1977; Drummond and Jones, 1979). Apnoea is the strongest trigger of bradycardia at the start of a dive (Foster and Sheel, 2005), although facial submersion can lead to bradycardia even when respiration is allowed to continue (Drummond and Jones, 1979; Foster and Sheel, 2005), and facial submersion and apnoea are synergistic for the development of bradycardia (Foster and Sheel, 2005). In fact, Irving *et al.* (1935) considered the dive response to be a modification of the physiological compensation during asphyxia. Apnoea results in changes in blood gas levels, and detection of these changes by the carotid bodies becomes increasingly important in maintaining bradycardia (de Burgh Daly *et al.*, 1977; Lin *et al.*, 1983). An additional effect of conscious awareness on bradycardia is suggested by two factors. Firstly, the degree of bradycardia during a dive response has been observed to increase when an animal is forcibly submerged or when an animal dives because it is exposed to a threatening stimulus (Butler and Jones, 1997). Secondly, anticipatory changes have been observed in some species, with bradycardia occurring or resolving shortly before diving or surfacing, respectively (Thompson and Fedak, 1993; Butler and Jones, 1997; Panneton, 2013). While afferent control by trigeminal nerve and carotid body stimulation is understood (de Burgh Daly *et al.*, 1977; Foster and Sheel, 2005; Panneton, 2013), and the efferent parasympathetic control of bradycardia and sympathetic control of compensatory vascular changes are known (Foster and Sheel, 2005; Panneton, 2013), there is less understanding of the central neural networks involved (Panneton, 2013). Given the number of stimuli involved and their changing relative effects, these neural networks are likely to be complex (Foster and Sheel, 2005).

Cardiac, respiratory and vascular changes similar to those of the dive response have been described in other situations, as a result of stimulation of the trigeminal nerve by mechanical or chemical receptors, e.g. in response to upper airway irritants (‘nasopharyngeal reflex’; Yu and Blessing, 1997), mechanical stimulation of ocular and periocular structures (‘oculocardiac reflex’; Schaller, 2004) or direct stimulation of any part of the sensory trigeminal nerve pathway (‘trigeminocardiac reflex’; Schaller *et al.*, 2009). Schaller (2004, p 663) considered these reflexes and the dive response to have ‘at least partially similar physiological mechanisms’, and Gorini *et al.* (2009, p 1443) described the dive response as ‘a subset of the trigeminocardiac reflex’. However, in the absence of a comprehensive understanding of the mechanisms of any of these events, it is not possible to know whether they are identical or merely similar responses to stimulation of the trigeminal nerve.

The platypus is found associated with inland water bodies in Eastern Australia and spends its time either feeding in water or resting in a burrow (Grant and Bishop, 1998). Platypuses are generally out of their burrow each day for a single period of 7.3–16 h (Serena, 1994; Gust and Handasyde, 1995; Otley *et al.*, 2000; Bethge, 2002). A platypus must dive to the bottom of the water body to find and collect its food (Grant, 2007), returning to the surface to breathe and masticate the food. Kruuk (1993) observed an average dive time of 35 s, with a maximum of 75 s in wild platypuses. Evans *et al.* (1994) found that in captive platypuses most dives lasted between 30 s and 4 min, but observed a maximal dive time of 11 min. Bethge (2002) observed a mean dive depth of 1.21 m and a maximal dive depth of 8.77 m in wild platypuses.

Two studies have investigated the dive response in platypuses. Johansen *et al.* (1966) forcibly submerged two platypuses tied to boards. Heart rates were observed to slow gradually over 30–35 s from pre-submersion levels of ∼140 beats/min to minimal values of ∼20 beats/min (Johansen *et al.*, 1966). Evans *et al.* (1994) used surgically implanted electrocardiogram/radio-transmitter systems to monitor the heart rate of five freely diving wild platypuses that had been brought into a captive situation. Pre-dive heart rates were in the range of 140–230 beats/min. Two patterns of heart rate changes were observed, associated with differing dive lengths. In dives of 1–3.5 min, during which the platypuses were usually inactive, the heart rate decreased over ∼30 s and remained stable at 12–40 beats/min. During dives of up to 1 min, heart rates were erratic but fell within seconds of diving to between 10 and 120 beats/min. Heart rates were generally lower during dives lasting 40–60 s (20–80 beats/min) than during dives lasting up to 30 s (70–120 beats/min; Evans *et al.*, 1994).

J. McKee (unpublished data) briefly described apnoea and bradycardia in anaesthetized platypuses. It was reported that these changes were likely to occur in individuals in a light plane of anaesthesia and usually resolved within 3 min without any reduction in anaesthetic dose. It was considered that an informal survey of four operators indicating that apnoea and bradycardia occurred in 1–2% of platypus inhalation anaesthetic procedures may have given an underestimate of the incidence, owing to the intermittent monitoring undertaken (J. McKee, unpublished data).

The aim of the present study was to examine the heart and respiratory rates of platypuses during isoflurane anaesthesia, currently the method of choice for platypus anaesthesia (Booth and Connolly, 2008; J. McKee, unpublished data), to identify periods of sudden-onset apnoea and bradycardia, to investigate factors by which they may be triggered and maintained and to evaluate whether the term ‘dive response’ is an appropriate description of these changes.

## Materials and methods

A total of 163 platypus field anaesthetic procedures were performed in the Inglis River catchment (41.06°S, 145.64°E) in Tasmania between August 2011 and August 2013 as part of a research project assessing the health of wild platypus populations. Platypuses were examined in the field close to the site of their capture either in a tent erected on site or in a farm shed (if available). After capture in a fyke net (Macgregor *et al*., 2010), each platypus was held in a cotton bag inside a hessian sack for a minimum of 1 h to allow its fur to dry and its stomach to empty (Booth and Connolly, 2008) before being anaesthetized for examination. When the air temperature was below 10°C, a 32–34°C hot water bottle was placed between the cotton bag and hessian sack in a position where the platypus could choose to be close to it or move away from it.

**Figure 1:**
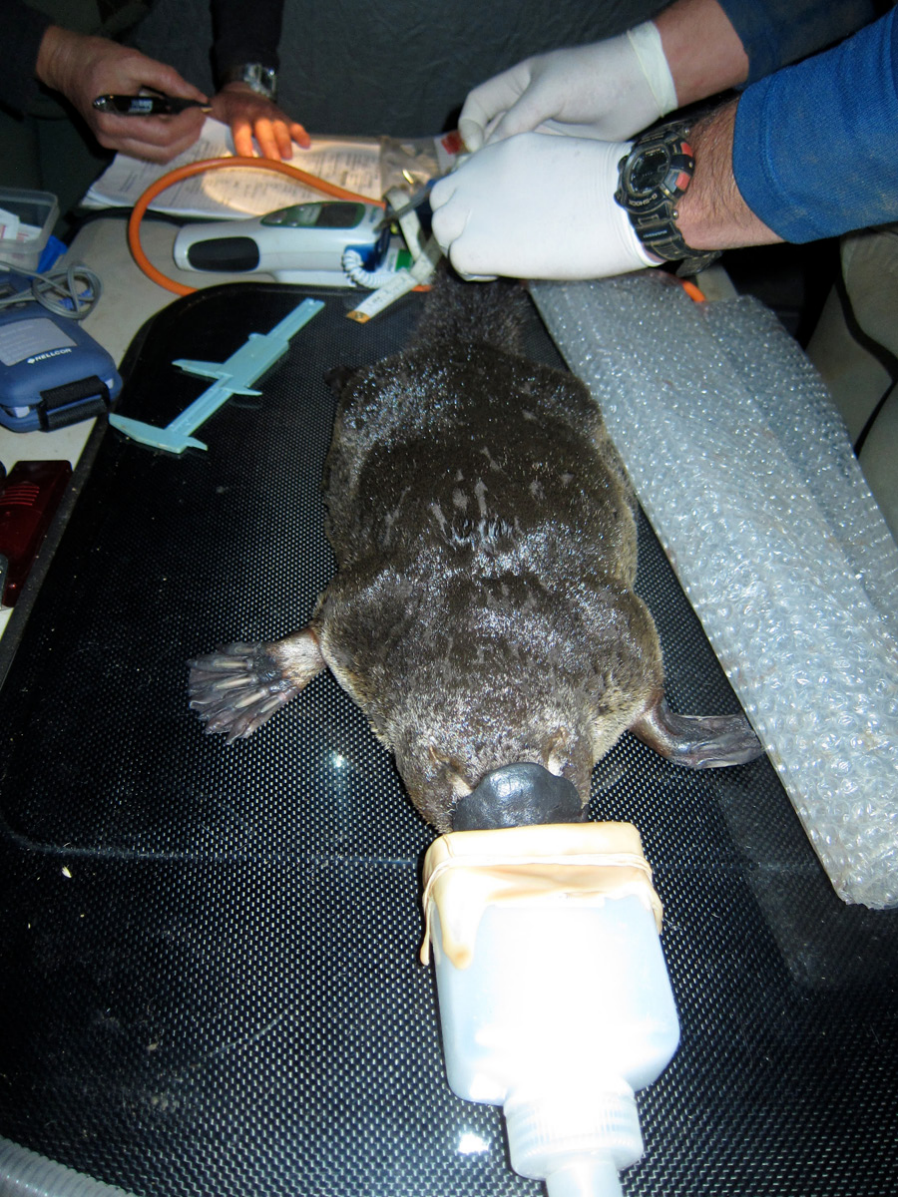
Mask anaesthesia of a platypus on a thermostatically controlled heat pad with a bubble wrap blanket (not yet in place) to regulate body temperature. Photograph by Christina Shaw.

Anaesthesia was induced with 5% isoflurane in oxygen at 2 l/min by face mask and was generally maintained with isoflurane at 1.5% in oxygen at 1.5 l/min. Body temperature was maintained by a thermostatically controlled heat pad and a bubble wrap blanket (Fig. 1), which were required due to the cool environmental temperatures associated with anaesthesia of platypuses in the field in Tasmania. The following parameters were assessed and recorded every 5 min: heart rate by auscultation, respiratory rate by visual examination, cloacal body temperature using a Welch Allyn SureTemp^®^ Plus 690 Electronic Thermometer (Welch Allyn, Skaneateles Falls, NY, USA) and anaesthetic depth by response to stimuli. The same parameters were also checked at varying frequencies between scheduled recordings while health examinations were being performed. To avoid pre-judging the nature of our observations, a new term, ‘sudden-onset apnoeic/bradycardic event’ (SOABE), was used to describe an anaesthetic response involving apnoea (no breath over >1 min) and bradycardia (<100 beats/min) lasting >1 min and occurring without any preceding or ongoing gradual decrease in heart rate and respiratory rate. Periods of apnoea and bradycardia lasting <1 min were regarded as transient events, which can be associated with anaesthesia in any animal. Observed gradual decreases in heart rate and/or respiratory rate were considered to be the result of anaesthetic dose-dependent respiratory and cardiac depression, and the isoflurane concentration was decreased. Heart and respiratory rates were monitored continuously or near continuously from the time an SOABE was suspected to the time eupnoea and eucardia were considered to have returned.

Student's unpaired *t*-tests were conducted to compare the minimal heart rate during SOABEs at induction and during recovery from anaesthesia and to compare the duration of SOABEs at induction and during recovery from anaesthesia (Statistica 8.0, Statsoft Inc., Tulsa, OK, USA). The occurrence of SOABEs at induction and during recovery (yes or no) was compared separately with intrinsic and extrinsic factors using forward stepwise logistic regression (Statistica 8.0, Statsoft Inc.). For occurrence at induction, factors investigated were season of capture (summer or winter; September–February or March–August), number of fieldworkers (as a measure of the potential noise levels around capture and handling time), whether the platypus was alone in the net when it was found (yes or no), whether the platypus had been captured previously (yes or no), whether the platypus was transported in a car (yes or no; if not then it would have been carried by hand in its holding sack from the capture site to the examination site), number of platypuses captured in the fieldwork session, sex (male or female; spur present or absent; Temple-Smith, 1973), the platypus's age (adult or non-adult; spur/spur sheath presence/morphology; Temple-Smith, 1973, as modified by Grant, 1991), the platypus's body condition (scale of 1 to 5; tail volume index; Grant and Carrick, 1978), the platypus's initial body temperature during anaesthesia (in degrees Celsius) and holding time before anaesthesia (in minutes). The relationships between occurrence of SOABE at recovery tested the same factors, with the addition of duration of isoflurane administration (in minutes) and estimated duration of dorsal recumbency for ultrasound examination (in minutes) based on ultrasound procedures performed (0 = no procedures, 2 = tail fat ultrasound only, 6 = abdominal ultrasound only and 8 = both tail fat and abdominal ultrasound).

Values are expressed as means ± 1 standard deviation.

## Results

Sudden-onset apnoeic/bradycardic events were never recorded during maintenance of anaesthesia, but were observed in 31 anaesthetized platypuses (19% of the 163 tested) during either induction (*n* = 9 individuals) or recovery (*n* = 17 individuals), or both (*n* = 5 individuals). Given that our monitoring was not continuous, the exact moments of transition from eupnoea to apnoea and from eucardia to bradycardia were not always observed. However, apnoea was observed to precede bradycardia on several occasions. On all other occasions, apnoea and bradycardia had both already started at the time when any changes were observed, and it was not possible to determine whether or not one preceded the other. Transient apnoea and bradycardia were observed at induction or recovery in an additional 17 platypuses. There were no platypus deaths during the fieldwork.

The typical range for heart rate during maintenance of stable anaesthesia was 114–162 beats/min. During a SOABE, sustained minimal heart rates were usually in the range 30–72 beats/min, but occasionally there was >10 s between two consecutive heart beats. The typical range for respiratory rate during maintenance of stable anaesthesia was 6–24 breaths/min. During a SOABE, respiration ceased for >1 min. After a period of ∼1–10 min of apnoea and bradycardia, heart rate would gradually rise before regular breathing recommenced. A short period (∼30–60 s) of tachycardia and tachypnoea would then occur, following which the apnoea and bradycardia would frequently return, usually with a slightly higher heart rate than previously. This process of apnoea and bradycardia interspersed with tachycardia and tachypnoea would continue for up to 20 min, with the periods of apnoea and bradycardia becoming steadily shorter and the bradycardia being less severe in successive periods, until eventually the platypus started to breathe regularly and the bradycardia ceased. The lowest recorded heart rate during SOABEs was significantly lower at induction (42 ± 21 beats/min) than for those recorded during recovery (61 ± 18 beats/min; *t*_33_ = −2.85, *P* = 0.007). There was no significant difference in the duration of SOABEs at induction (4 ± 3 min) and during recovery (11 ± 7 min; *t*_10_ = −1.77, *P* = 0.107). During SOABEs at induction, platypuses generally retained some degree of voluntary movement and withdrawal reflex, but when the SOABE ceased they quickly stabilized and became non-responsive under anaesthesia. At recovery, some platypuses that had shown initial signs of waking up became less alert and less responsive to stimulation after the onset of a SOABE. When the event ceased, the platypuses awakened quickly.

**Figure 2:**
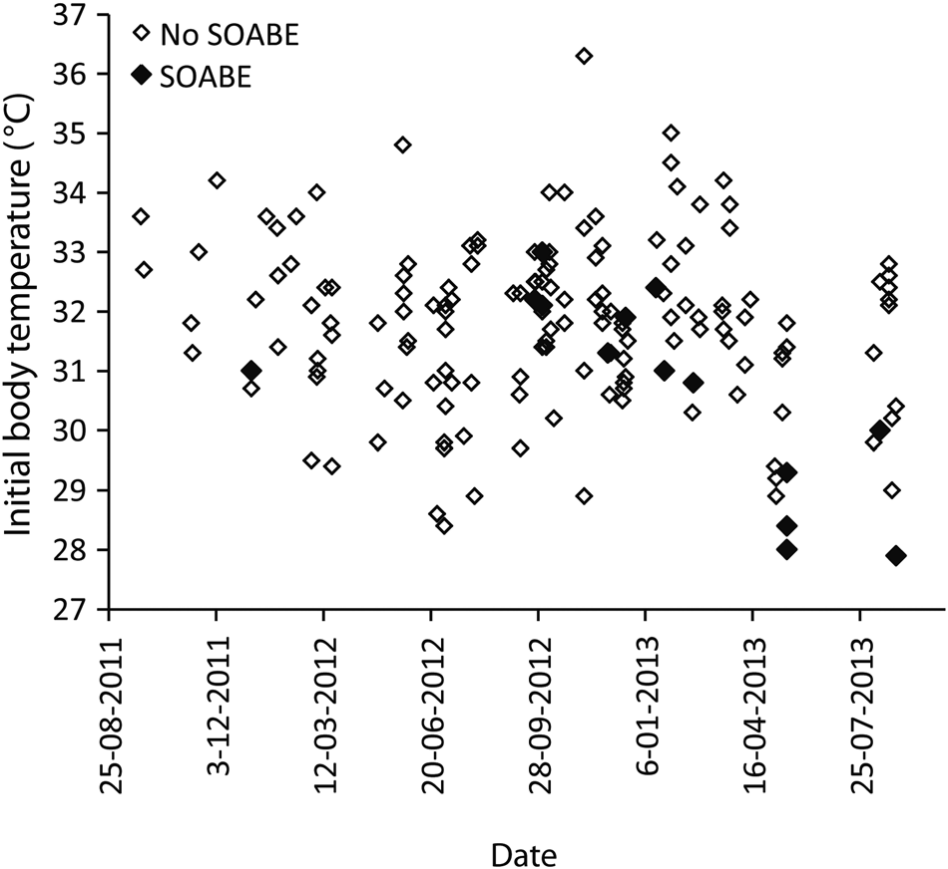
Occurrence of sudden-onset apnoeic/bradycardic event (SOABE) at induction of anaesthesia in 163 platypuses, shown by date and initial body temperature.

**Figure 3:**
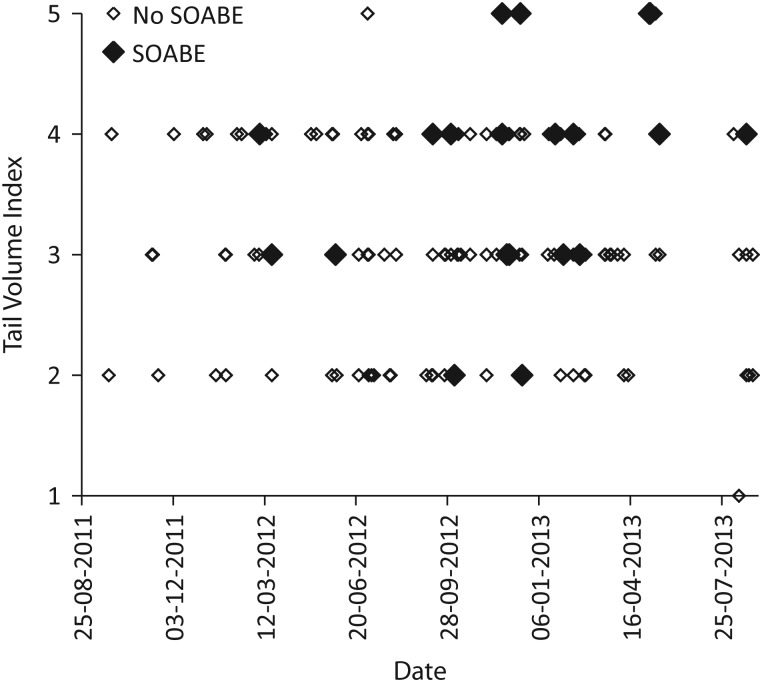
Occurrence of SOABE at recovery from anaesthesia in 163 platypuses, shown by date and tail volume index as a measure of body condition (5 = poorest body condition, 1 = best body condition).

**Figure 4:**
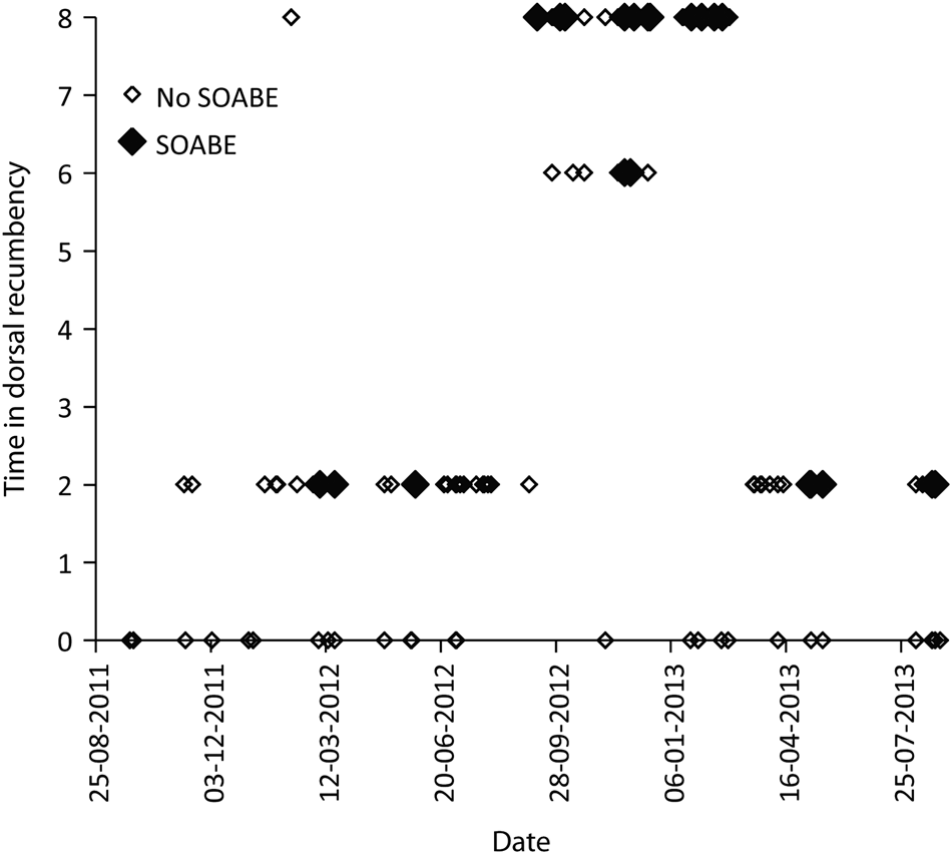
Occurrence of SOABE at recovery from anaesthesia in 163 platypuses, shown by date and time in dorsal recumbency (see Materials and methods section for time estimation details).

Of the 163 anaesthetic procedures, 67 were performed on females (64 adults and three juveniles) and 96 were performed on males (84 adults and 12 juveniles/subadults). Other intrinsic and extrinsic factors of these platypuses are summarized in Tables 1 and 2. The results from the forward stepwise regression (Table 3) revealed that the occurrence of a SOABE at induction correlated with low body temperature (*P* < 0.001). Although they did not yield statistically significant values, three other factors were also included in the final model; there was a greater occurrence in summer (*P* = 0.06) and in adults (*P* = 0.19), and there was a positive correlation with increasing pre-anaesthetic holding time (*P* = 0.16). Three of the four platypuses which had the lowest initial core body temperatures (<28.5°C) shortly after induction all had a SOABE at induction (Fig. 2). The remaining 11 platypuses with SOABEs at induction had body temperatures between 30 and 34°C. No platypuses with initial body temperature >34°C had a SOABE at induction. Sudden-onset apnoeic/bradycardic events at recovery occurred only in platypuses that had been placed in dorsal recumbency as part of their examination. Their occurrence correlated with time in dorsal recumbency (*P* = 0.005) and poor body condition (*P* = 0.002) in the forward stepwise regression model (Figs 3 and 4; Table 3). Although they did not yield statistically significant values, three other factors were also included in this stepwise model; there was a positive correlation with the number of fieldworkers (*P* = 0.06), and SOABEs were more frequent in adults (*P* = 0.06) and in females (*P* = 0.11).

**Table 1:** Summary of categorical intrinsic and extrinsic factors relating to anaesthetized platypuses

	Yes	No
Capture occurred in summer	85	78
Platypus alone in net when it was found	135	28
Platypus had been captured previously	12	151
Platypus transported in a car	70	93

**Table 2:** Summary of continuous intrinsic and extrinsic factors relating to anaesthetized platypuses

	Mean	SD	Maximum	Minimum
Number of fieldworkers	3.1	1.0	6	2
Number of platypuses captured in the session	2.5	1.3	5	1
Body condition index (tail volume index)	3.2	0.9	5	1
Initial body temperature during anaesthesia (°C)	31.7	1.4	36.3	27.9
Holding time before anaesthesia (min)	124.7	49.5	324	60
Duration of isoflurane anaesthesia (min)	25.1	6.0	40	9
Duration of dorsal recumbency for ultrasound examination (min)	3.8	3.2	8	0

**Table 3:** Summary of forward stepwise logistic regression for factors correlated with the occurrence of a sudden-onset apnoeic/bradycardic event at induction and during recovery

	Induction	Recovery
Season of capture (summer or winter)	*P* = 0.06	–
Number of fieldworkers (count)	–	*P* = 0.06
Platypus alone in the net when it was found (yes or no)	–	–
Platypus had been captured previously (yes or no)	–	–
Platypus transported in a car (yes or no)	–	–
Number of platypuses captured in the fieldwork session (count)	–	–
Age (juvenile or adult)	*P* = 0.19	*P* = 0.06
Sex (male or female)	–	*P* = 0.11
Body condition (tail volume index)	–	*P* = 0.002
Initial body temperature during anaesthesia (°C)	*P* < 0.001	–
Holding time before anaesthesia (min)	*P* = 0.16	–
Duration of isoflurane administration (min)	NA	–
Duration of dorsal recumbency for ultrasound examination (min)	NA	*P* = 0.005

NA indicates factors that were not considered for the occurrence of a sudden-onset apnoeic/bradycardic event at induction; – indicates a factor that did not improve the model during the stepwise analysis and was not included in the final model.

## Discussion

In the following discussion, we will show that the respiratory and cardiac characteristics of the SOABE we have described in platypuses during isoflurane anaesthesia are consistent not only with the dive response, but also with certain other previously described responses. We will propose that the mechanisms that trigger and maintain the SOABE during anaesthesia are similar to the control mechanisms that regulate the dive response but that there is a different primary trigger. Consequently, we will suggest a change in nomenclature for this response. We will also discuss additional factors associated with the incidence of SOABEs in platypuses and discuss measures that can be taken during future studies to reduce their effects.

The SOABEs observed during anaesthetic induction or recovery in platypuses were consistent with those previously seen in voluntarily diving platypuses, forcibly submerged platypuses and naturally diving seals (Table 4; Johansen *et al.*, 1966; Jones *et al.*, 1973; Evans *et al.*, 1994). They were also consistent with the changes recorded in rabbits during anaesthetic induction (Table 5; Flecknell *et al.*, 1996). Although blood pressure and blood gas analysis were beyond the scope of our project, the observations of these parameters by Flecknell *et al.* (1999) in rabbits were similar to those observed during diving in marine mammals. Flecknell *et al.* (1999; p 45) concluded that the mechanism for the apnoea, bradycardia, blood gas changes and blood pressure changes they observed during induction of isoflurane anaesthesia in rabbits appeared ‘to be similar to that associated with breathholding in man’.

**Table 4:** Characteristics of apnoea and bradycardia in voluntarily diving platypuses, forcibly submerged platypuses and naturally diving seals and platypuses during isoflurane anaesthesia

	Voluntary dives of 40–60 s in platypuses in holding facility (Evans *et al.*, 1994)	Forcibly submerged platypuses (Johansen *et al.*, 1966)	Naturally diving harbour seals (Jones *et al.*, 1973)	Platypuses undergoing mask induction with isoflurane (present study)
Timing of bradycardia in relationship to apnoea	Immediate onset, peaked after 15 s	Bradycardia started 5–10 s after apnoea and peaked after 30–35 s	Bradycardia started 2–3 s after submergence	Sudden onset. Apnoea observed before bradycardia on occasion, but never the reverse
Minimal heart rate (% of initial heart rate)	∼20	∼12–37	∼40–50	∼30–45

**Table 5:** Characteristics of the apnoea and bradycardia in rabbits and platypuses during isoflurane anaesthesia

	Rabbits undergoing face mask-delivered isoflurane anaesthesia (Flecknell *et al.*, 1996)	Platypuses undergoing face mask-delivered isoflurane anaesthesia (present study)
Timing of apnoea/bradycardia	Induction	Induction and/or recovery
Onset of apnoea	Isoflurane concentration >0.5%	∼4 min after start of induction or recovery
Minimal heart rate (% of resting heart rate)	18–45	At induction, ∼30; at recovery, ∼45
Duration of first period of apnoea and bradycardia (s)	30–120 s	60–600 s
Multiple periods of apnoea and bradycardia interspersed with short periods of tachypnoea and tachycardia	Yes	Yes
Level of consciousness during apnoea/bradycardia	Attempted to escape and pawed at nose	At induction, eyes often open, withdrawal reflexes intact, occasional spontaneous movements of head and legs. At recovery, often no movement but sometimes eyes open, withdrawal reflexes intact and/or arched back
Proportion of animals affected	100%	9% at induction 13% at recovery

**Figure 5:**
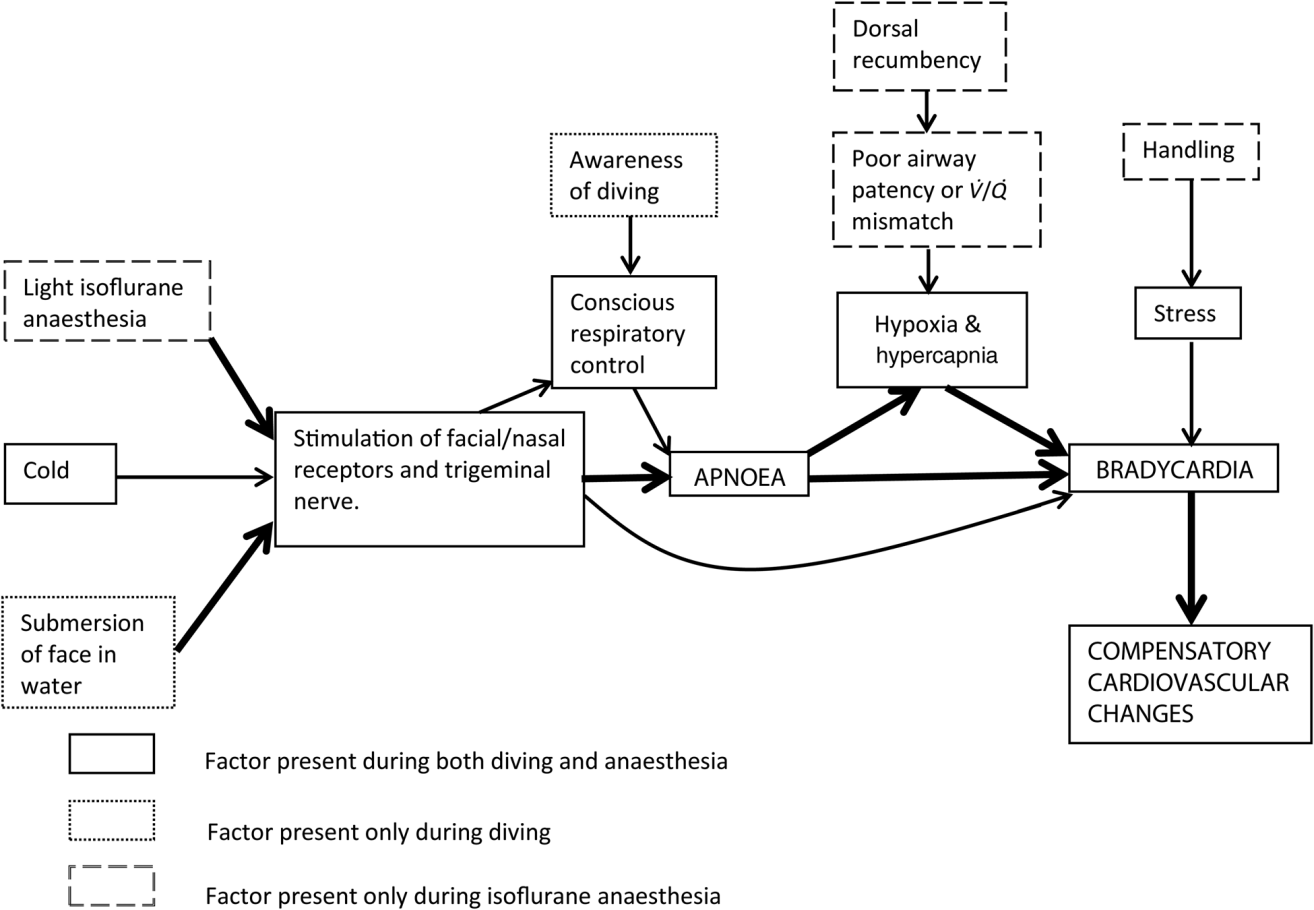
Path diagram for the development and maintenance of apnoea and bradycardia during diving and during isoflurane anaesthesia in the platypus. Abbreviation: *V·*/*Q·*, ventilation–perfusion. Bold arrows indicate stronger effects.

Isoflurane is known to cause airway irritation in a number of species and can lead to increased airway secretions as well as coughing and, most importantly in relationship to the SOABEs we observed, breath-holding during light anaesthesia (Clarke and Trim, 2013). The degree to which this breath-holding is a conscious response is not known; however, it has been shown that exposure of nasal receptors to airborne irritants in rabbits can, particularly in the conscious animal, trigger apnoea and bradycardia as a result of trigeminal nerve stimulation (Yu and Blessing, 1997). We hypothesize that in certain susceptible species, including the rabbit and the platypus, stimulation of nasal receptors and the trigeminal nerve by isoflurane may lead not only to apnoea, but also to bradycardia, either as a secondary response to the apnoea or directly from stimulation of the nasal receptors (Fig. 5).

Although we believe that nasal irritation by isoflurane was necessary for development of the SOABEs we observed, our results indicate that the onset and maintenance of a SOABE was also influenced by other factors. Likewise, there appear to be factors other than facial submersion and apnoea that affect the onset and characteristics of the cardiovascular changes during diving. For example, Jones *et al.* (1973) observed that one harbour seal (*Phoca vitulina richardi*) showed no bradycardia in 20% of its feeding dives. Also, observed heart rates in forcibly submerged platypuses and inactive voluntarily diving platypuses in a holding facility have been observed to be lower than those in active platypuses undertaking shorter voluntary dives (Johansen *et al.*, 1966; Evans *et al.*, 1994).

We acknowledge that in our study not all SOABEs may have been recorded because our monitoring was not continuous and that our definition of a SOABE may have excluded some events that resulted from the action of isoflurane on nasal chemoreceptors. However, we are confident that no anaesthetic dose-dependent apnoeic/bradycardic events were misclassified as SOABEs, because at induction the bradycardia did not continue to deteriorate when respiration occurred despite maintained isoflurane levels, and at recovery the apnoea and bradycardia occurred after isoflurane delivery had ceased.

Sudden-onset apnoeic/bradycardic events were more likely to happen during induction if the initial body temperature of the platypus was low. This was heavily influenced by the fact that three of the four platypuses with the lowest initial core body temperatures shortly after induction all had a SOABE at induction. The remaining 11 platypuses showing SOABE at induction had body temperatures between 30 and 34°C; Grigg *et al.* (1992) found 98.5% of temperature recordings from freely ranging platypuses in winter in the Thredbo River to be within the same limits. The occurrence of SOABEs in platypuses with apparently normal core body temperatures is consistent with the idea that low temperature was only one of a number of trigger factors for the SOABEs we observed. Alternatively, it may be that core body temperature appeared to influence the rate of occurrence only because it was a proxy for the facial/nasal temperature, but that it was not a good one. Cold ambient temperature was a common feature of our fieldwork, owing to the geographical location. While we made attempts to maintain platypus body temperature in order to avoid hypothermia, we were cautious because hyperthermia is also a risk during platypus handling and anaesthesia (J. McKee, unpublished data), particularly in captivity and warm environmental conditions.

Sudden-onset apnoeic/bradycardic events during recovery from anaesthesia occurred only in platypuses that had been placed in dorsal recumbency as part of their examination, and the rate of occurrence was related to the duration of dorsal recumbency. Respiratory movements of the platypuses during dorsal recumbency appeared to be exaggerated, and it may be that either poor upper airway patency or ventilation–perfusion (*V·*/*Q·*) mismatching in the lungs lead to increasing levels of hypoxia and hypercapnia. Hypoxia and hypercapnia have been observed to be involved in the development and maintenance of a natural dive response (de Burgh Daly *et al.*, 1977; Lin *et al.*, 1983); they may also have been important triggers for the apnoea and bradycardia that we observed. They may have been particularly important during recovery, when the nasal isoflurane concentration would be expected to be lower than at induction, and when we had usually either partly or completely corrected any hypothermia present at induction. However, blood gas changes would also have occurred at induction when apnoea occurred, and if they were involved in developing and maintaining apnoea and bradycardia during recovery, they are likely to have had the same actions during induction. Other factors which may have resulted in the occurrence of a SOABE could be associated with an effect of increased stress levels in individuals at the time of anaesthesia in a similar manner to the effects observed in mammals and birds that dive naturally in response to threatening stimuli (Butler and Jones, 1997).

Two aspects of our results have no parallel during diving. The first of these is the cyclical nature of the apnoea and bradycardia. This was also seen during rabbit anaesthesia (Flecknell *et al.*, 1996) and provides strong evidence that the same phenomenon was being observed in the two species. It is likely that in the platypus the first period of tachycardia and tachypnoea occurs at roughly the time when the conscious animal would no longer be able to breath-hold during a dive and would come to the surface. The triggers for the break point of breath-holding are complex and not fully understood (Parkes, 2006). Prolongation of breath-holding times in humans with no apparent peripheral or central blood gas chemoreceptivity provides evidence that, in individuals with blood gas chemoreceptivity, hypoxia and hypercapnia are involved with break point occurrence (Parkes, 2006). In general, however, the blood gas levels at break point are inconsistent between repeat breath-holds or with inspiration of varying levels of oxygen or carbon dioxide before breath-holding (Parkes, 2006). If blood gas pressures were the sole determinants of the break point timing, it would not be possible to make a second breath-hold until these parameters had been improved. However, again in humans, Fowler (1954) demonstrated that allowing eight breaths of an asphyxiating gas mixture (8% oxygen, 7.5% carbon dioxide) at the break point of breath-holding allowed a second and then a third 20 s breath-hold to be performed. It has also been shown that a second breath-hold is possible if respiratory effort occurs against a closed airway and no gas is taken into the lungs (Rigg *et al.*, 1974). There may be a role for diaphragmatic chemoreceptors and/or proprioceptors as well as lung volume and metabolic rate in determining the maximal breath-hold duration (Parkes, 2006). In particular, removal of diaphragmatic receptor stimulation may be the reason that a second breath-hold is possible despite ongoing hypoxia and hypercapnia (Parkes, 2006). Regardless of the exact mechanism of breath-hold break point determination, it seems plausible that if the triggers that initiated apnoea and bradycardia in isoflurane-anaesthetized platypuses remain after the stimuli to breathe have been relieved, apnoea and bradycardia will recur.

The second observation that cannot be related to the naturally diving animal is that some platypuses became less alert and less responsive to stimulation after the onset of a SOABE during recovery. Given that the trigeminal nerve-mediated cardiovascular and respiratory responses are largely under parasympathetic control, this reduced responsiveness could have a similar mechanism to a freeze response, which can occur as a response to fear, also under parasympathetic control (Hermans *et al.*, 2013).

We propose that, with the exception of transient anticipatory changes that have been described at the beginning and end of diving (Butler and Jones, 1997; Panneton, 2013), the overall mechanism of trigeminal nerve stimulation leading to apnoea and, either directly or secondarily to apnoea, to bradycardia (de Burgh Daly *et al.*, 1977), applies to the changes occurring in the platypus during natural diving and during light isoflurane anaesthesia. This mechanism is shown in Fig. 5, which is in part a summary of the conclusions of previous research into the dive response. However, given that the primary trigger for trigeminal nerve stimulation under isoflurane anaesthesia is the presence of an irritant gas and not the presence of water in the nasal cavities, we propose that the term ‘nasopharyngeal response’ would more appropriately describe the changes we observed. We consider it likely that the mechanism in Fig. 5 will also apply to other diving animals during isoflurane anaesthesia. However, given that we observed no platypus deaths and given the protective physiological nature of the responses to apnoea and trigeminal nerve stimulation discussed, the apnoea/bradycardia-associated deaths reported by McDonell (1972), Gales and Burton (1988) and Phelan and Green (1992) would not be expected from such a response (Lynch *et al.*, 1999). Marine mammal anaesthesia poses a number of challenges, including narrow anaesthetic agent dose ranges, a large amount of peripharyngeal tissue that can reduce airway patency, and the flexible nature of the thorax that requires considerable inspiratory effort to act against the weight of the thorax (Hammond and Elsner, 1977; Gage, 2003; Tahmindjis *et al.*, 2003). It seems likely that the marine mammal deaths that have been reported to have been associated with apnoea and bradycardia have been at least in part associated with these other anaesthetic complications, as reported by Tahmindjis *et al.* (2003). Apnoea and bradycardia have been reported in marine mammals as a consequence of injectable anaesthesia, during which nasal chemoreceptor stimulation would be absent. Seals can show periodic breathing, with the heart rate slowing during 20–40 s periods of apnoea (Thompson and Fedak, 1993), or can undergo physiological bradycardia during periods of apnoea of up to 20 min while resting on land (Andrews *et al.*, 1997), with similarities to the bradycardia observed during voluntary breath-holding and asphyxia in other species (Bauer, 1938; Bjurström and Schoene, 1987). Given the considerable ability of diving mammals to endure apnoea (Irving, 1939; Kooyman *et al.*, 1981), these species presumably have physiological mechanisms enhanced for this purpose, and it would not be surprising if apnoea during marine mammal anaesthesia leads to bradycardia more readily than in terrestrial animals.

Further research is indicated to clarify the roles of stress, temperature and blood gases on the development of sudden-onset apnoea and bradycardia during isoflurane anaesthesia in platypuses. However, a lower frequency of what we can now refer to as the nasopharyngeal response during platypus anaesthesia might be achieved by one or more of the following: use of a less irritant anaesthetic gas, such as sevoflurane; premedication with injectable sedative(s); not placing platypuses in dorsal recumbency; further increasing efforts to mitigate stress on captured platypuses; and warming and humidifying the inhaled gas.

## Funding

This work was supported by Winifred Violet Scott Estate, a Caring For Our Country Community Action Grant [Project ID CAG 11-00128], the Central North Field Naturalists, the National Geographic Society [Grant C217-12], the Cradle Coast Natural Resource Management, Tasmanian Alkaloids, the Australian Geographic Society, the Forestry Practices Authority, and the Edward Alexander Weston and Iris Evelyn Fernie Research Fund.

## References

[COU053C1] Andrews R Jones D Williams J Thorson P Oliver G Costa D Le Boeuf B (1997) Heart rates of northern elephant seals diving at sea and resting on the beach. J Exp Biol 200: 2083–2095. 9255950 10.1242/jeb.200.15.2083

[COU053C2] Bauer D (1938) The effect of asphyxia upon the heart rate of rabbits at different ages. J Physiol 93: 90–103. 16995002 10.1113/jphysiol.1938.sp003628 PMC1393724

[COU053C3] Bethge P (2002) Energetics and foraging behaviour of the platypus, *Ornithorhynchus anatinus*. PhD thesis University of Tasmania, Hobart, Tasmania, Australia.

[COU053C4] Bjurström RL Schoene RB (1987) Control of ventilation in elite synchronized swimmers J Appl Physiol 63: 1019–1024. 3654451 10.1152/jappl.1987.63.3.1019

[COU053C5] Booth R Connolly J (2008) Platypuses. In Vogelnest L Woods R, eds, Medicine of Australian Mammals. CSIRO Publishing, Collingwood, Victoria, Australia, pp 103–132.

[COU053C6] Butler PJ Jones DR (1997) Physiology of diving of birds and mammals. Physiol Rev 77: 837–899. 9234967 10.1152/physrev.1997.77.3.837

[COU053C7] Clarke KW Trim CM (2013) Veterinary Anaesthesia. Elsevier Health Sciences, Amsterdam, The Netherlands.

[COU053C8] de Burgh Daly M Elsner R Angell-James JE (1977) Cardiorespiratory control by carotid chemoreceptors during experimental dives in the seal. Am J Physiol Heart Circ Physiol 232: H508–H516. 10.1152/ajpheart.1977.232.5.H508 860766

[COU053C9] Drummond P Jones D (1979) The initiation and maintenance of bradycardia in a diving mammal, the muskrat, *Ondatra zibethica*. J Physiol 290: 253–271. 469759 10.1113/jphysiol.1979.sp012770 PMC1278834

[COU053C10] Evans B Jones D Baldwin J Gabbott G (1994) Diving ability of the platypus. Aust J Zool 42: 17–27.

[COU053C11] Flecknell P Cruz I Liles J Whelan G (1996) Induction of anaesthesia with halothane and isoflurane in the rabbit: a comparison of the use of a face-mask or an anaesthetic chamber. Lab Anim 30: 67–74. 8709576 10.1258/002367796780744910

[COU053C12] Flecknell P Roughan J Hedenqvist P (1999) Induction of anaesthesia with sevoflurane and isoflurane in the rabbit. Lab Anim 33: 41–46. 10759391 10.1258/002367799780578516

[COU053C13] Foster G Sheel A (2005) The human diving response, its function, and its control. Scand J Med Sci Sports 15: 3–12. 15679566 10.1111/j.1600-0838.2005.00440.x

[COU053C14] Fowler WS (1954) Breaking point of breath-holding. J Appl Physiol 6: 539–545. 13152037 10.1152/jappl.1954.6.9.539

[COU053C15] Gage L (2003) Pinnipedia. In Fowler M Miller R eds, Zoo and Wild Animal Medicine, Ed 5 WB Saunders Co., St Louis, MO, USA, pp 459–475.

[COU053C16] Gales N Burton H (1988) Use of emetics and anesthesia for dietary assessment of Weddell seals. Wildlife Res 15: 423–433.

[COU053C17] Gorini C Jameson HS Mendelowitz D (2009) Serotonergic modulation of the trigeminocardiac reflex neurotransmission to cardiac vagal neurons in the nucleus ambiguus. J Neurophysiol 102: 1443–1450. 19553488 10.1152/jn.00287.2009 PMC2746775

[COU053C18] Grant T (2007) Platypus. CSIRO Publishing, Collingwood, Victoria, Australia.

[COU053C19] Grant T Bishop K (1998) Instream flow requirements for the platypus (*Ornithorhynchus anatinus*): a review. Aust Mammal 20: 267–280.

[COU053C48] Grant T Carrick F (1978) Some aspects of the ecology of the platypus, *Ornithorhynchus anatinus*, in the Upper Shoalhaven River, New South Wales. Aust Zool 20: 181–199.

[COU053C20] Grigg G Beard L Grant T Augee M (1992) Body-temperature and diurnal activity patterns in the platypus (*Ornithorhynchus anatinus*) during winter. Aust J Zool 40: 135–142.

[COU053C21] Gust N Handasyde K (1995) Seasonal-variation in the ranging behavior of the platypus (Ornithorhynchus-anatinus) on the Goulburn River, Victoria. Aust J Zool 43: 193–208.

[COU053C22] Hammond D Elsner R (1977) Anesthesia in phocid seals. J Zoo Anim Med 8: 7–13.

[COU053C23] Hermans EJ Henckens MJ Roelofs K Fernández G (2013) Fear bradycardia and activation of the human periaqueductal grey. Neuroimage 66: 278–287. 23110885 10.1016/j.neuroimage.2012.10.063

[COU053C24] Irving L (1939) Respiration in diving mammals Physiol Rev 19: 112–134.

[COU053C25] Irving L Solandt O Solandt D Fisher K (1935) The respiratory metabolism of the seal and its adjustment to diving. J Cell Comp Physiol 7: 137–151.

[COU053C26] Irving L Scholander P Grinnell S (1941) Significance of the heart rate to the diving ability of seals. J Cell Comp Physiol 18: 283–297.

[COU053C27] Johansen K Lenfant C Grigg G (1966) Respiratory properties of blood and responses to diving of the platypus, *ornithorhynchus anatinus* (Shaw). Comp Biochem Physiol 18: 597–608. 5967684 10.1016/0010-406x(66)90243-x

[COU053C28] Jones DR Fisher HD McTaggart S West NH (1973) Heart rate during breath-holding and diving in the unrestrained harbor seal (Phoca vitulina richardi). Can J Zool 51: 671–680. 4756147 10.1139/z73-101

[COU053C29] Kooyman G Castellini M Davis R (1981) Physiology of diving in marine mammals. Annu Rev Physiol 43: 343–356. 7011189 10.1146/annurev.ph.43.030181.002015

[COU053C30] Kruuk H (1993) The diving behaviour of the platypus (*Ornithorhynchus anatinus*) in waters with different trophic status. J Appl Ecol 30: 592–598.

[COU053C31] Lin Y Shida K Hong S (1983) Effects of hypercapnia, hypoxia, and rebreathing on heart rate response during apnea. J Appl Physiol 54: 166–171. 6402469 10.1152/jappl.1983.54.1.166

[COU053C32] Lynch M Tahmindjis M Gardner H (1999) Immobilisation of pinniped species. Aust Vet J 77: 181–185. 10197248 10.1111/j.1751-0813.1999.tb11231.x

[COU053C47] Macgregor J Holyoake C Munks S Robertson I Warren K (2010) Preliminary investigation into the prevalence of mucormycosis in the platypus (*Ornithorhynchus anatinus*) in three catchments in north-west Tasmania. Aust Vet J 88: 190–196. 20529030 10.1111/j.1751-0813.2010.00568.x

[COU053C33] McDonell W (1972) Anesthesia of the harp seal. J Wildl Dis 8: 287–295. 5049122 10.7589/0090-3558-8.3.287

[COU053C35] Otley HM Munks SA Hindell MA (2000) Activity patterns, movements and burrows of platypuses (*Ornithorhynchus anatinus*) in a sub-alpine Tasmanian lake. Aust J Zool 48: 701–713.

[COU053C36] Panneton WM (2013) The mammalian diving response: an enigmatic reflex to preserve life? Physiology (Bethesda) 28: 284–297. 23997188 10.1152/physiol.00020.2013 PMC3768097

[COU053C37] Parkes M (2006) Breath-holding and its breakpoint. Exp Physiol 91: 1–15. 16272264 10.1113/expphysiol.2005.031625

[COU053C38] Phelan J Green K (1992) Chemical restraint of Weddell seals (*Leptonychotes weddellii*) with a combination of tiletamine and zolazepam. J Wildl Dis 28: 230–235. 1602573 10.7589/0090-3558-28.2.230

[COU053C39] Rigg J Rebuck A Campbell E (1974) A study of factors influencing relief of discomfort in breath-holding in normal subjects. Clin Sci Mol Med 47: 193–199. 4425035 10.1042/cs0470193

[COU053C40] Schaller B (2004) Trigeminocardiac reflex. J Neurol 251: 658–665. 15311339 10.1007/s00415-004-0458-4

[COU053C41] Schaller B Cornelius JF Prabhakar H Koerbel A Gnanalingham K Sandu N Ottaviani G Filis A Buchfelder M (2009) The trigemino-cardiac reflex: an update of the current knowledge. J Neurosurg Anesthesiol 21: 187–195. 19542994 10.1097/ANA.0b013e3181a2bf22

[COU053C42] Serena M (1994) Use of time and space by platypus (*Ornithorhynchus anatinus*: Monotremata) along a Victorian stream. J Zool 232: 117–131.

[COU053C43] Tahmindjis MA Higgins DP Lynch MJ Barnes JA Southwell CJ (2003) Use of a pethidine and midazolam combination for the reversible sedation of crabeater seals (*Lobodon carcinophagus*). Mar Mam Sci 19: 581–589.

[COU053C49] Temple-Smith PD (1973) Seasonal breeding biology of the platypus, *Ornithorhynchus anatinus* (Shaw, 1799), with special reference to the male. PhD thesis, Australian National University, Canberra.

[COU053C44] Thompson D Fedak MA (1993) Cardiac responses of grey seals during diving at sea. J Exp Biol 174: 139–154. 8440964 10.1242/jeb.174.1.139

[COU053C45] Yu Y-H Blessing W (1997) Cerebral blood flow in rabbits during the nasopharyngeal reflex elicited by inhalation of noxious vapor. J Auton Nerv Syst 66: 149–153. 9406119 10.1016/s0165-1838(97)00080-5

[COU053C46] Zapol WM Liggins G Schneider R Qvist J Snider MT Creasy RK Hochachka PW (1979) Regional blood flow during simulated diving in the conscious Weddell seal. J Appl Physiol 47: 968–973. 511722 10.1152/jappl.1979.47.5.968

